# Single-nuclei transcriptomics reveals cellular senescence in pediatric anthracycline-induced cardiomyopathy

**DOI:** 10.1172/JCI204909

**Published:** 2026-08-25

**Authors:** Diwakar Turaga, Chang-Ru Tsai, Yuka Morikawa, Hanna J. Tadros, Yi Zhao, Lalita Wadhwa, Iki Adachi, Xiao Li, James F. Martin

**Affiliations:** 1 Department of Pediatrics, Baylor College of Medicine, Houston, Texas, USA.; 2 Division of Critical Care Medicine, Texas Children’s Hospital, Houston, Texas, USA.; 3 Department of Integrative Physiology and; 4 Texas Heart Institute, Baylor College of Medicine, Houston, Texas, USA.; 5 Division of Cardiology, Texas Children’s Hospital, Houston, Texas, USA.; 6 Department of Surgery, Baylor College of Medicine, Houston, Texas, USA.; 7 Division of Congenital Heart Surgery, Texas Children’s Hospital, Houston, Texas, USA.; 8 Department of Molecular and Human Genetics and; 9 Center for Organ Repair and Renewal, Baylor College of Medicine, Houston, Texas, USA.

**Keywords:** Cardiology, Oncology, Heart failure

## Abstract

Chemotherapy drugs that fight childhood cancer can age heart cells prematurely. We found this “cellular aging” throughout the hearts of children with treatment-related heart failure.

**To the Editor:** Anthracyclines such as doxorubicin are integral to pediatric cancer treatment, yet their use carries a substantial long-term cardiovascular burden. Pediatric anthracycline-induced cardiomyopathy is a major late effect, with progression to advanced heart failure and the need for transplantation in a subset of survivors (1). Although anthracycline cardiotoxicity has been extensively studied in experimental models and adult populations (2, 3), the developing pediatric myocardium, characterized by distinct metabolic states, stress-adaptive responses, and cell-cycle regulation, may respond differently to chemotoxic injury. As a result, the in vivo, cell type–specific transcriptional consequences of anthracycline exposure in children remain poorly defined, underscoring the need for direct interrogation of human pediatric myocardial tissue.

We performed single-nuclei RNA sequencing (snRNA-seq) on left ventricular myocardium to generate a high-resolution, cell type–resolved transcriptomic atlas of pediatric anthracycline-induced cardiomyopathy (pAICM). We integrated data from 5 female pediatric patients exposed to anthracyclines who progressed to end-stage heart failure requiring transplantation, along with age- and sex-matched patients with pediatric dilated cardiomyopathy (pDCM) with end-stage heart failure and nonfailing pediatric donors (pDonors) (Figure 1A and Supplemental Table 1; supplemental material available online with this article; https://doi.org/10.1172/JCI204909DS1). Following quality control and integration, the final dataset comprised 63,826 nuclei from patients with pAICM, 31,973 from patients with pDCM, and 29,190 from pDonors (Figure 1B). Unsupervised clustering identified 11 major cardiac cell populations, annotated using established canonical markers, with cell type composition varying across groups (Figure 1C and Supplemental Figure 1, A–D).Kyoto Encyclopedia of Genes and Genomes pathway analysis in pAICM cardiomyocytes revealed selective enrichment of the senescence pathway (Figure 1D). Cellular senescence is a stress-induced cell fate characterized by stable cell-cycle arrest and a proinflammatory, hypersecretory phenotype (4). We then applied a curated composite gene set integrating canonical senescence pathways and senescence-associated secretory phenotype (SASP) components (Senescence Signature Genes; Figure 1A). Using this signature, pAICM cardiomyocytes demonstrated significantly higher senescence scores compared with pDCM and pDonor cardiomyocytes (*P* < 0.0001; Figure 1E). Elevated senescence scores were also observed in pAICM-derived endothelial cells, fibroblasts, and myeloid cells (*P* < 0.0001 for all), relative to cells derived from pDCM and pDonor (Figure 1E), indicating this is not explained by end-stage systolic dysfunction alone.

Consistent with these global transcriptional changes, key senescence-associated genes (including *ANGPTL4*, *STAT3*, *JUN*, and *CDKN1A*) were selectively upregulated in pAICM cardiomyocytes (Figure 1F). Immunostaining for p21, a canonical mediator of senescence-associated cell-cycle arrest encoded by *CDKN1A*, demonstrated robust nuclear expression in pAICM myocardium, with minimal staining in pDonor tissue (Figure 1G). Quantification of cardiomyocyte p21 positivity, aggregated at the disease level, revealed a marked increase in the proportion of p21^+^ cardiomyocytes in the pAICM group compared with that in donor controls (*P* <0.001; Figure 1H).

Applying our senescence signature to a published adult AICM dataset (5) revealed no substantial elevation in senescence scores across major cell types relative to adult donors, suggesting that multicellular senescence may represent a pediatric-specific response to anthracycline cardiotoxicity (Supplemental Figure 1, E and F). Subcluster analysis across pAICM cardiomyocyte, endothelial cell, fibroblast, and myeloid populations revealed that senescence scores were elevated uniformly across all cell states, supporting a pan-cellular senescence phenotype (Supplemental Figure 2). Differential gene expression and Gene Ontology enrichment analysis comparing cells derived from patients with pAICM with pDonor cells and cells from patients with pDCM revealed distinct pathway signatures across cell types, underscoring the broad transcriptional effect of anthracycline exposure on the pediatric myocardium (Supplemental Figure 3).

In summary, we identified increased cellular senescence across multiple cardiac cell types in pAICM. While these data do not establish causality, the absence of comparable senescence activation in age-matched patients with pDCM and adult patients with AICM supports a pediatric anthracycline-specific effect. A major limitation of this study is the small, female-only cohort, reflecting the extreme rarity of pediatric transplant myocardium with documented anthracycline exposure. Nonetheless, end-stage human pediatric tissue offers otherwise unattainable insight and establishes a resource for future mechanistic and therapeutic studies in childhood cancer survivors.

For detailed methods, information regarding sex as a biological variable, statistics, study approval, author contributions, acknowledgments, and data availability, see the supplemental materials.

## Conflict of interest

JFM is a cofounder and owns shares in Medley Therapeutics.

## Funding support

This work is the result of NIH funding, in whole or in part, and is subject to the NIH Public Access Policy. Through acceptance of this federal funding, the NIH has been given a right to make the work publicly available in PubMed Central.

- NIH grants R01HL127717, R01HL118761, R01HL171574, R01HL169511 to JFM.
- NIH grant R01HL179012 to XL.
- Additional Ventures Catalyst to Independence Award to DT.
- Vivian L. Smith Foundation to JFM.
- Don McGill Gene Editing Laboratory of the Texas Heart Institute at Baylor College of Medicine to XL.

## Supplementary Material

Supplemental data

Supporting data values

## Figures and Tables

**Figure 1:**
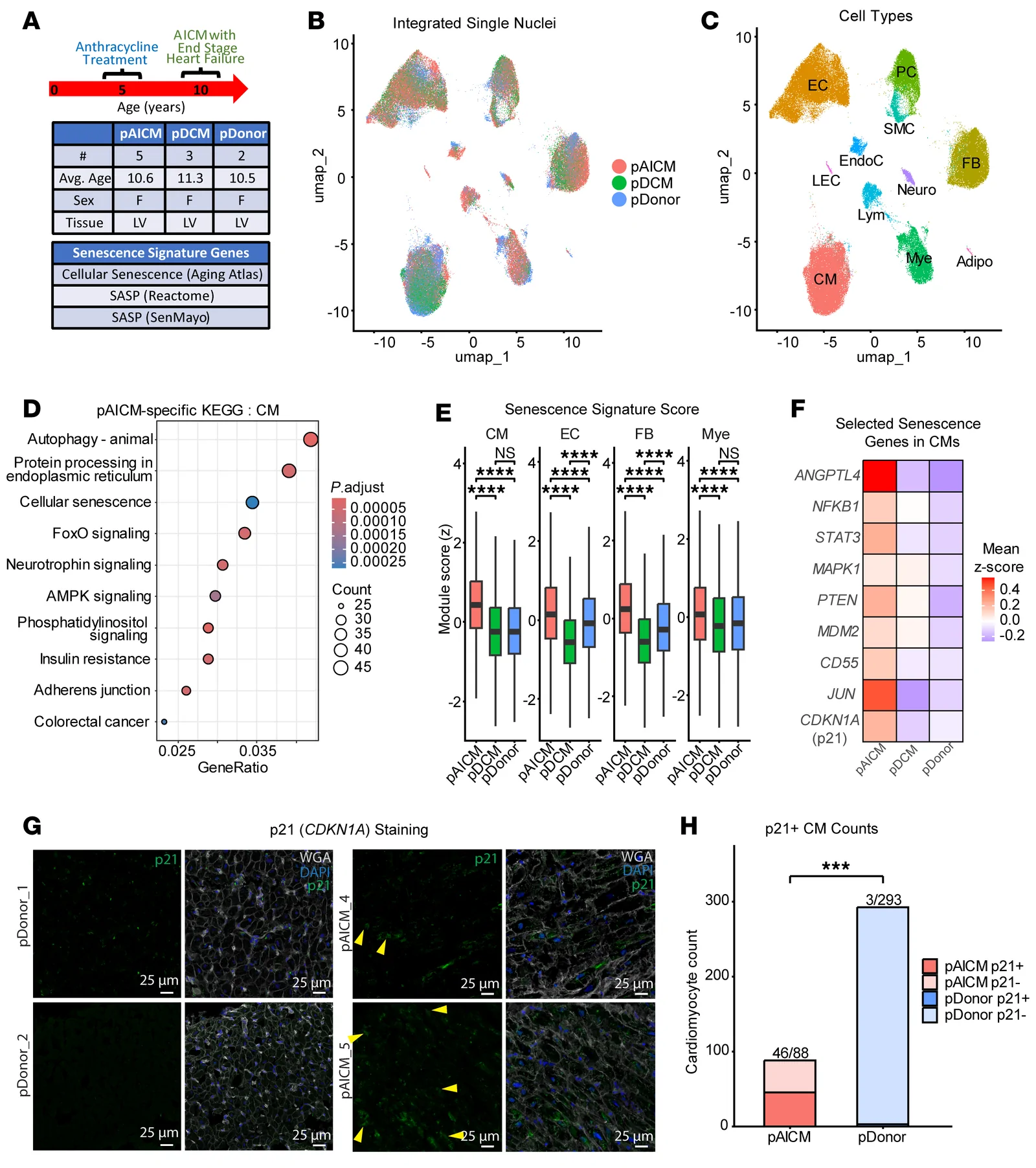
Cellular senescence in pediatric anthracycline-induced cardiomyopathy. (**A**) Clinical timeline and patient demographics for pediatric anthracycline-induced cardiomyopathy (pAICM) samples, with senescence gene sets used for analyses. (**B**) Batch-corrected UMAP of nuclei from pAICM, pediatric dilated cardiomyopathy (pDCM), and pediatric donor (pDonor) hearts. (**C**) Major cardiac cell populations identified by unsupervised clustering and canonical markers. (**D**) KEGG pathway analysis of pAICM cardiomyocyte-specific genes showing enrichment of cellular senescence pathway. (**E**) Senescence signature scores across major cardiac cell types, demonstrating increased senescence signature in pAICM samples compared with pDCM and pDonor samples. (Wilcoxon’s rank-sum test with Benjamini-Hochberg FDR correction; ****P < 0.0001). (**F**) Mean cardiomyocyte expression of representative senescence-associated genes. (**G**) Representative immunofluorescence staining of pAICM and pDonor myocardium for p21 (green), wheat germ agglutinin (WGA; white), and DAPI (blue). Arrowheads indicate p21^+^ nuclei. Scale bar: 25 μm. (**H**) Quantification of p21^+^ cardiomyocytes in pAICM and pDonor hearts, aggregated across 2 independent fields of view per individual and 2 patients per disease group. Bars represent disease groups; the bracket indicates the comparison between pAICM and pDonor. Counts above each bar denote p21^+^ cardiomyocytes out of total cardiomyocytes. (Fisher’s exact test on pooled cardiomyocyte counts; ***P < 0.001). LV, left ventricle; UMAP, Uniform Manifold Approximation and Projection; CM, cardiomyocytes; EC, endothelial cells; FB, fibroblasts; PC, pericytes; Mye, myeloid cells; SMC, smooth muscle cells; Lym, lymphoid cells; EndoC, endocardial cells; Neuro, neuronal cells; LEC, lymphatic endothelial cells; Adipo, adipocytes; KEGG, Kyoto Encyclopedia of Genes and Genomes.

## Version 1 08/25/2026

In-Press Preview

## Version 2 10/15/2026

Electronic publication

## References

[1] Ryan TD (2025). Cardiovascular toxicity in patients treated for childhood cancer: a scientific statement from the American Heart Association. Circulation.

[2] Ghigo A (2025). Update on preclinical models of cancer therapy-related cardiac dysfunction: Challenges and perspectives. A scientific statement of the Heart Failure Association (HFA) of the ESC, the ESC Council of Cardio-Oncology, and the ESC Working Group on Cellular Biology of the Heart. Eur J Heart Fail.

[3] Camilli M (2024). Anthracycline cardiotoxicity in adult cancer patients: *JACC: CardioOncology* State-of-the-Art Review. JACC CardioOncol.

[4] Chen MS (2022). Senescence mechanisms and targets in the heart. Cardiovasc Res.

[5] Guo Z (2025). Human single-nucleus RNA sequencing identifies CD47 as a therapeutic target for doxorubicin-induced cardiomyopathy. Circulation.

