# Supplementary material for: Single-nuclei transcriptomics reveals cellular senescence in pediatric anthracycline-induced cardiomyopathy
Source: J Clin Invest. 2026 Aug 25;136(20):e204909. doi: 10.1172/JCI204909 (PMC13668626; doi:10.1172/JCI204909)
Supplement: Supplementary file 1 [file jci-136-204909-s140.pdf]

METHODS AND SUPPLEMENTAL INFORMATION

Research Ethics for Donated Tissues

Cardiac tissue samples were obtained during clinically indicated cardiothoracic surgical procedures at Texas Children's Hospital (Houston, TX). Procurement and use of human myocardial specimens were approved by the Institutional Review Board of Baylor College of Medicine and Affiliated Hospitals (Protocol H-26502). In collaboration with the Texas Children's Hospital Heart Center Biorepository, written informed consent was obtained from patients or legal guardians prior to heart transplantation or ventricular assist device implantation.

Sex as a biological variable

All pediatric anthracycline-induced cardiomyopathy (pAICM) patients included in this study were female, consistent with prior reports indicating increased susceptibility to anthracycline-associated cardiotoxicity in females (1, 2). To minimize confounding related to sex-specific differences in cardiac biology, comparator groups were restricted to female non-failing pediatric donors (pDonors) and pediatric dilated cardiomyopathy (pDCM) samples.

Publicly Available Data

Pediatric datasets: Previously published cardiac snRNA-seq data from pediatric donors were obtained from the NIH Gene Expression Omnibus (GEO) as non-failing controls. These datasets include three libraries from donor "UK1" reported by Hill et al. (GSE203275) and one library from donor "13_235" by Koenig et al. (GSE183852) (3, 4). All samples originated from the left ventricles of female donors.

Sample Collection and Nuclear Isolation

Cardiac tissue from the left ventricle (large mid-myocardial biopsy) was collected intraoperatively during heart transplantation and immediately placed in cold saline on ice for transport to the laboratory. Upon

receipt, tissue was carefully dissected into multiple aliquots, flash-frozen, and stored at −80 °C until processing. Nuclei were isolated using the 10x Genomics Chromium Nuclei Isolation Kit (PN-100494) according to the manufacturer's protocol. Briefly, frozen cardiac tissue was mechanically dissociated, followed by sequential centrifugation steps to remove debris and enrich for high-quality nuclei.

Single-nuclei RNA Sequencing

Single-nuclei RNA sequencing (snRNA-seq) was performed using the 10x Genomics Chromium platform. Isolated nuclei were loaded onto the Chromium Controller to generate gel bead–in–emulsion. Sequencing libraries were prepared according to the manufacturer's protocol using the Single Cell 3′ Reagent Kits v3. Libraries were sequenced on an Illumina NovaSeq 6000 system.

snRNA-seq Data Processing and Integration

All newly generated and previously published pediatric snRNA-seq datasets were processed using a unified analysis pipeline as described previously (5). Briefly, raw sequencing reads were aligned to the human reference genome (GRCh38) using the 10x Genomics Cell Ranger toolkit (v7.1.0; cellranger count) with the --include-introns option enabled; all other parameters were set to default. Standard Cell Ranger quality control metrics were reviewed for each library.

To remove ambient RNA contamination, raw UMI count matrices were further processed using CellBender (v0.2.2; remove-background) with the following parameters: --total-droplets-included=25,000, --low-count-threshold=15, and --epochs=200. To minimize loss of valid nuclei identified by Cell Ranger, the --expected-cells parameter was set to 1.5× the number of nuclei reported by Cell Ranger. CellBender output matrices were subsequently filtered to retain only barcodes also identified by Cell Ranger.

Additional nucleus-level quality control was performed for each library. Low-quality nuclei were initially excluded using fixed thresholds of >200 UMIs per nucleus, >150 detected genes per nucleus, and <5%

mitochondrial gene–derived UMIs. To account for inter-sample heterogeneity, dynamic, library-specific thresholds were then applied: upper and lower bounds for UMI and gene counts were defined as the 75th percentile plus 1.5× the interquartile range (IQR) and the 25th percentile minus 1.5× IQR, respectively.

Potential doublets were identified using Scrublet with an expected doublet rate of 0.15 and a doublet score threshold of 0.25 (6). Finally, all samples were integrated, and batch effects were corrected using the probabilistic modeling tool, scVI. For scVI integration, 3,000 most variable genes were first selected across libraries. The raw UMI count matrix of these highly variable genes across all cells were modeled with scVI, accounting for batch effects as well as library- and donor-level covariates. Additional continuous covariates, including total UMI counts, detected feature numbers, and mitochondrial UMI percentages, were incorporated to improve normalization and batch correction. The scVI model was trained with a two-layer neural network and a latent dimensionality of 50, optimized over 400 training epochs. The resulting 50-dimensional latent representation was used consistently for downstream analyses, including neighborhood graph construction, clustering, and UMAP visualization.

Clustering and Annotation

Cell clustering was performed using the Seurat package (v4.2.0). A shared nearest-neighbor (SNN) graph was constructed using the FindNeighbors function applied to the scVI latent space. Clusters were identified from the SNN graph using the Louvain algorithm with a resolution parameter of 1.0. Because additional scVI model was trained using the annotated reference dataset from Litviňuková *et al.*(7), predicted cell-type labels were examined for each cluster. Final cell-type annotations were assigned based on concordance between scANVI-predicted labels and the expression of established cardiac cell-type marker genes.

Pathway enrichment analysis

Differential gene expression analysis between pediatric anthracycline-induced cardiomyopathy (pAICM) and non-failing pediatric donor cardiomyocytes (pDonors) was performed using the Seurat package. Marker genes were identified using the Wilcoxon rank-sum test (FindMarkers), requiring expression in at least 10% of cells in either group and a minimum log2 fold change of 0.25. *P* values were adjusted for multiple testing using the Benjamini–Hochberg false discovery rate (FDR) method. Genes with an FDR-adjusted *P* value < 0.05 and increased expression in pAICM were considered significantly upregulated and selected for downstream analyses.

Significantly upregulated pAICM genes were used for pathway enrichment analyses. Gene symbols were converted to Entrez Gene identifiers using the bitr function from the clusterProfiler package with the org.Hs.eg.db annotation database. Kyoto Encyclopedia of Genes and Genomes (KEGG) pathway enrichment analysis was performed using the enrichKEGG function, specifying *Homo sapiens* as the reference organism. Pathways were considered significantly enriched based on Benjamini–Hochberg false discovery rate–adjusted *P* values < 0.05. Enrichment results were visualized using dot plots displaying the top enriched pathways.

Genes significantly upregulated in pAICM relative to both pDonor and pDCM (adjusted P < 0.05, log2 fold-change > 0.25) within each major cell type were defined as pAICM-specific and used for Gene Ontology (GO) biological process enrichment analysis. GO analysis was performed using the enrichGO function from the clusterProfiler package, with gene symbols provided directly as input (keyType = "SYMBOL") against the org.Hs.eg.db human annotation database. Enrichment significance was assessed using a Benjamini–Hochberg false discovery rate adjustment; terms with adjusted P < 0.05 and q-value < 0.2 were considered significantly enriched. The top 10 enriched terms ranked by GeneRatio are displayed as dot plots, where dot size reflects the number of contributing genes and dot color reflects the adjusted p-value.

Gene Set Expression Scoring

Gene set–based scoring was performed using the AddModuleScore function in the Seurat package. This approach implements a previously described method (8) in which each cell is assigned a score calculated as the average expression of genes in a specified gene set, normalized by subtracting the average expression of a matched background gene set. For each gene in the target set, 100 background genes with similar average expression levels across all cells were randomly selected. Module scores were subsequently scaled and centered to generate Z-scores.

Senescence signature scores were derived from curated gene sets obtained from public databases, including the Reactome Pathway Database (R-HSA-2559583) and AgeingAtlas. Senescence-associated secretory phenotype (SASP) scores were calculated using the previously published SenMayo gene set (9).

Tissue Immunohistochemistry

A limitation of our study is the restricted tissue availability: immunohistochemistry could be performed only on pAICM_4 and pAICM_5, as suitable tissue was not available from the remaining pAICM patients. Immunohistochemistry on cardiac tissues was performed on OCT embedded frozen sections. Sections were post-fixed in 10% NBF, treated with 0.3% H_2O_2 in PBS then 5 mM ammonium chloride in PBS, then permeabilized with 0.5% Tween 20 in PBS, for 10 min each treatment. After staining with 10% donkey serum in PBST (PBS + 0.1% Tween 20) for 1 hr, tissues were incubated with 1:200 anti-p21 antibody (Abcam, ab188224) in 10% donkey serum in PBST overnight at 4˚C. After washing with PBST, sections were incubated with HRP linked anti-rabbit IgG, HRP (Thermo Fisher Scientific, 31458) for 10 min at room temperature. After washing with PBST, tissues were incubated with 1:200 Opal 520 reagent (Akoya Biosciences, OP-001001) in 1X plus amplification diluent (Akoya Biosciences, FP1498) for 10 min. After washing in PBST, sections were stained with DAPI and WGA Alexa Fluor 555 (Thermo

Fisher Scientific, W32464) in PBS for 10 min. Immunostained slides were mounted with Dako Fluorescence mounting media (Agilent). Mounted sections were imaged using a Nikon AX confocal microscope. Nuclei and cell boundaries were visualized using DAPI and WGA, respectively. Cardiomyocytes were identified based on morphological features in the WGA channel. Cardiomyocytes were classified as p21-positive when nuclear p21 signal intensity exceeded that of the cytoplasm within the same cell. p21 positivity was scored manually by an observer blinded to group assignment, across two fields of view per patient.

STATISTICS:

Figure 1D: Differential gene expression was performed within the specified cell type using Seurat (v5). The comparison was conducted between pAICM vs pDonor using two-sided Wilcoxon rank-sum tests (FindMarkers, test.use = "wilcox"), requiring expression in at least 10% of cells (min.pct = 0.1) and an absolute log2 fold-change threshold of 0.25 (logfc.threshold = 0.25). P values were adjusted for multiple testing across genes using the Benjamini–Hochberg method, and genes with adjusted $P < 0.05$ and positive log2 fold change were considered upregulated. Functional enrichment analyses were performed on the resulting gene list after conversion to Entrez identifiers (clusterProfiler/AnnotationDbi). KEGG pathway enrichment was tested using hypergeometric over-representation analysis (enrichKEGG) with Benjamini–Hochberg adjustment; pathways with $q < 0.05$ were considered significant.

Figure 1E: Senescence gene set module scores were calculated at the single-cell level for each cell type separately using Seurat AddModuleScore with a predefined senescence gene set. Module scores were standardized by z transformation within each cell type prior to analysis. Group differences were assessed independently within each cell type using two-sided Wilcoxon rank-sum tests for pairwise comparisons among pAICM, pDCM, and pDonor. Within each cell type, P values were corrected for multiple comparisons across the three pairwise tests using the Benjamini-Hochberg false discovery rate procedure.

Significance is displayed as symbols: NS, not significant; *P < 0.05; **P < 0.01; ***P < 0.001; ****P < 0.0001.

Figure 1H: Cardiomyocyte p21 positivity was analyzed as a binomial outcome. For each patient, p21-positive and total cardiomyocyte counts were aggregated across two independent fields of view, then pooled across the two patients within each disease group. The resulting p21-positive and p21-negative counts were compared between pAICM and pDonor using a two-sided Fisher's exact test.

Supplementary Figure 1C: Cell type composition was calculated as the percentage of total nuclei assigned to each major cell type within each group (pAICM, pDCM, pDonor) and displayed as stacked bar plots.

Supplementary Figure 1D: Per-patient cell type proportions were calculated as the percentage of total nuclei per sample assigned to each of the four major cell compartments (CM, EC, FB, Mye). Each data point represents an individual patient sample.

Supplementary Figure 1E: Senescence gene set module scores were calculated using Seurat AddModuleScore applied separately to the adult AICM dataset (Guo et al.) for each major cell type. Group differences were assessed using two-sided Wilcoxon rank-sum tests for pairwise comparisons among aAICM, aDCM, and aDonor within each cell type. Within each cell type, P values were corrected for multiple comparisons across the three pairwise tests using the Benjamini-Hochberg false discovery rate procedure. Significance is displayed as symbols: NS, not significant; *P < 0.05; **P < 0.01; ***P < 0.001; ****P < 0.0001.

Supplementary Figure 1F: Delta mean senescence z-score was calculated as the difference between the mean normalized senescence score of each disease group and its respective donor control (pDonor for pediatric groups; aDonor for adult groups). Values are displayed as grouped bar plots across CM, EC, FB, and Mye compartments for pAICM vs. pDonor, pDCM vs. pDonor, aAICM vs. aDonor, and aDCM vs. aDonor.

Supplementary Figure 2B, E, H, K: Cell state composition was calculated as the percentage of total nuclei assigned to each cell state within each group (pAICM, pDCM, pDonor) and displayed as stacked bar plots.

Supplementary Figure 2C, F, I, L: Senescence gene set module scores were calculated at the single-cell level within each cell type subcluster using Seurat AddModuleScore with the predefined senescence gene set. Module scores were standardized by z-transformation within each cell state prior to analysis. Group differences were assessed independently within each cell state using two-sided Wilcoxon rank-sum tests for pairwise comparisons among pAICM, pDCM, and pDonor. Within each cell state, P values were corrected for multiple comparisons across the three pairwise tests using the Benjamini-Hochberg false discovery rate procedure. Significance is displayed as symbols: NS, not significant; *P < 0.05; **P < 0.01; ***P < 0.001; ****P < 0.0001.

Supplementary Figure 3A, B, C, D: Differential gene expression was performed within each major cell type (CM, EC, FB, Mye) using Seurat (v5) FindMarkers with two-sided Wilcoxon rank-sum tests, requiring expression in at least 10% of cells (min.pct = 0.1) and an absolute log2 fold-change threshold of 0.25 (logfc.threshold = 0.25). P values were adjusted for multiple testing using the Benjamini–Hochberg method; genes with adjusted P < 0.05 and positive log2 fold-change were considered significantly upregulated. Two comparisons were performed per cell type: pAICM vs. pDonor and pAICM vs. pDCM. Upregulated genes were stratified into three categories: unique to pAICM vs. pDonor, unique to pAICM vs. pDCM, and common to both comparisons. Genes common to both comparisons were defined as pAICM-specific and used for downstream pathway enrichment analysis.

Supplementary Figure 3E, F, G, H: Gene Ontology (GO) biological process enrichment analysis was performed on pAICM-specific upregulated genes (common to both comparisons) for each cell type using enrichGO (clusterProfiler v4, org.Hs.eg.db) with gene symbols as input (keyType = "SYMBOL"), Benjamini–Hochberg p-value adjustment, and significance thresholds of adjusted P < 0.05 and q < 0.2. The top 10 enriched terms ranked by GeneRatio are displayed. Dot size represents the number of genes contributing to each term (Count); dot color reflects the adjusted p-value.

Study Approval

Procurement and use of human myocardial specimens were reviewed and approved by the Institutional Review Board of Baylor College of Medicine and Affiliated Hospitals (Houston, TX: Protocol H-26502). Written informed consent was obtained from all patients or their legal guardians prior to participation in the study.

Author Contributions

DT: Conceptualization, methodology, investigation, formal analysis, visualization, funding acquisition, writing – original draft, writing – review & editing. CT: Investigation. YM: Investigation. HJT: Resources. YZ: Formal analysis. LW: Resources. IA: Resources. XL: Formal analysis, funding acquisition. JFM: Conceptualization, supervision, project administration, funding acquisition, writing – review & editing

Data Availability

Values for all data points shown in graphs and reported in the manuscript are provided in the Supporting Data Values file.

The processed single-cell object file is deposited in the EU Open Research Repository: https://zenodo.org/. It can be accessed via the following link: https://zenodo.org/records/20055673?token=eyJhbGciOiJIUzUxMiJ9.eyJpZCI6ImUxNTc3N2JlLWU4Y2QtNDE4ZS04ZmU1LTMyYjRjOTliMDM3ZiIsImRhdGEiOnt9LCJyYW5kb20iOiJkMmY3ZmIyY2JkYjRmZjIxNmNiN2FiYzFiMzBmMTUwMSJ9.xJz_kArA8fsajQ3JZ421tvS_UidCksh8efDQGPKnwkXrnHw_JOQU-KNiK2-PHcNmVrePUwJw3Mgrnuto7qjKvQ

Acknowledgments

The statistical analyses presented in this work were reviewed by Duy M. Dinh, PhD, Senior Biostatistician, Division of Critical Care Medicine, Texas Children's Hospital, whose input we gratefully acknowledge.

Supplementary Table 1: Clinical, echocardiographic, and histopathological characteristics of pAICM, pDCM, and pDonor subjects included in the study.

Sample	pAICM_1	pAICM_2	pAICM_3	pAICM_4	pAICM_5
ID	P73	P201	P283	P116	P126
Age (yrs)	13	11	14	11	4
Sex	F	F	F	F	F
Oncology Diagnosis	AML	AML	AML	Osteosarcoma	Congenital AML
Age at Diagnosis (yrs)	4	6	5	10	0
Treatment	AAML0531	AAML1031	AAML1131	AOST0331	AAML1031
Anthracycline (mg/m2)	490	338	490	450	300
Additional treatments	None	None	BMT @ 9 yrs	RUE amputation	None
LVEF (%)	23	25	19	31	29
LVEDD (cm)	7.9	5.9	5	7.2	4.5
LVEDD (z score)	0.3	3.6	1.3	6	-0.8
LVESD (cm)	7.5	5.4	4.8	6.5	3.7
LV Mass/Height^2.7 (g/m)	47	66	46	73	66

Sample	pDCM_1	pDCM_2	pDCM_3	pDonor_1	pDonor_2
ID	P32	P154	P87	UK1_FC3CB	13_235
Age (yrs)	10	11	13	10	11
Sex	F	F	F	F	F
Diagnosis	DCM	DCM	DCM/LVNC		
Genetics	Invitae: Neg	Invitae: TTN	Invitae: MYH7, GAA		
Data Source				Hill et al., Nature, 2022.	Koenig et al., Nature CVR, 2022.
LVEF (%)	16	21	31		
LVEDD (cm)	5.7	6.2	8.1		
LVEDD (z score)	4.1	2.9	10.7		
LVESD (cm)	4.8	5.8	6.9		
LV Mass/Height^2.7 (g/m)	54	58	57		

Sample	Pathology
pAICM_1	Moderate (focally marked) diffuse myocardial hypertrophy with mild patchy interstitial and pericellular fibrosis, focal myofibrillar loss, and small degenerative mitochondria with mild polymorphism.
pAICM_2	Endocardial fibrosis with nearly diffuse subendocardial vacuolar myocytolysis, interstitial perimyocyte and replacement fibrosis, and variably small mitochondria.
pAICM_3	Multifocal interstitial replacement fibrosis with focally accentuated subendocardial fibrosis and focal myocytolysis.
pAICM_4	Extensive cardiomyocyte degeneration with myocytolysis, small-to-large vacuolization, and myofibrillar loss. Moderate-to-marked myocyte hypertrophy with patchy interstitial fibrosis.
pAICM_5	Mild-to-moderate myocyte hypertrophy with marked endocardial fibrous thickening. Increased mitochondria without significant pleomorphism.
pDCM_1	Marked myocyte hypertrophy with mild endocardial thickening (no significant fibroelastosis), patchy mild-to-moderate interstitial and replacement fibrosis, focal interstitial and perivascular lymphocytic infiltrate, and mild mitochondrial pleomorphism.
pDCM_2	Marked myocyte hypertrophy with pericellular fibrosis and vacuolar myocytolysis. No mitochondrial pleomorphism or increased mitochondrial numbers.
pDCM_3	Mild myocyte hypertrophy without interstitial fibrosis.

Abbreviations: AML, acute myeloid leukemia; BMT, bone marrow transplantation; RUE, right upper extremity; LVNC, left ventricular non-compaction; LVEF, left ventricular ejection fraction; LVEDD, left ventricular end-diastolic dimension; LVESD, left ventricular end-systolic dimension; LV, left ventricle; F, female; yrs, years.

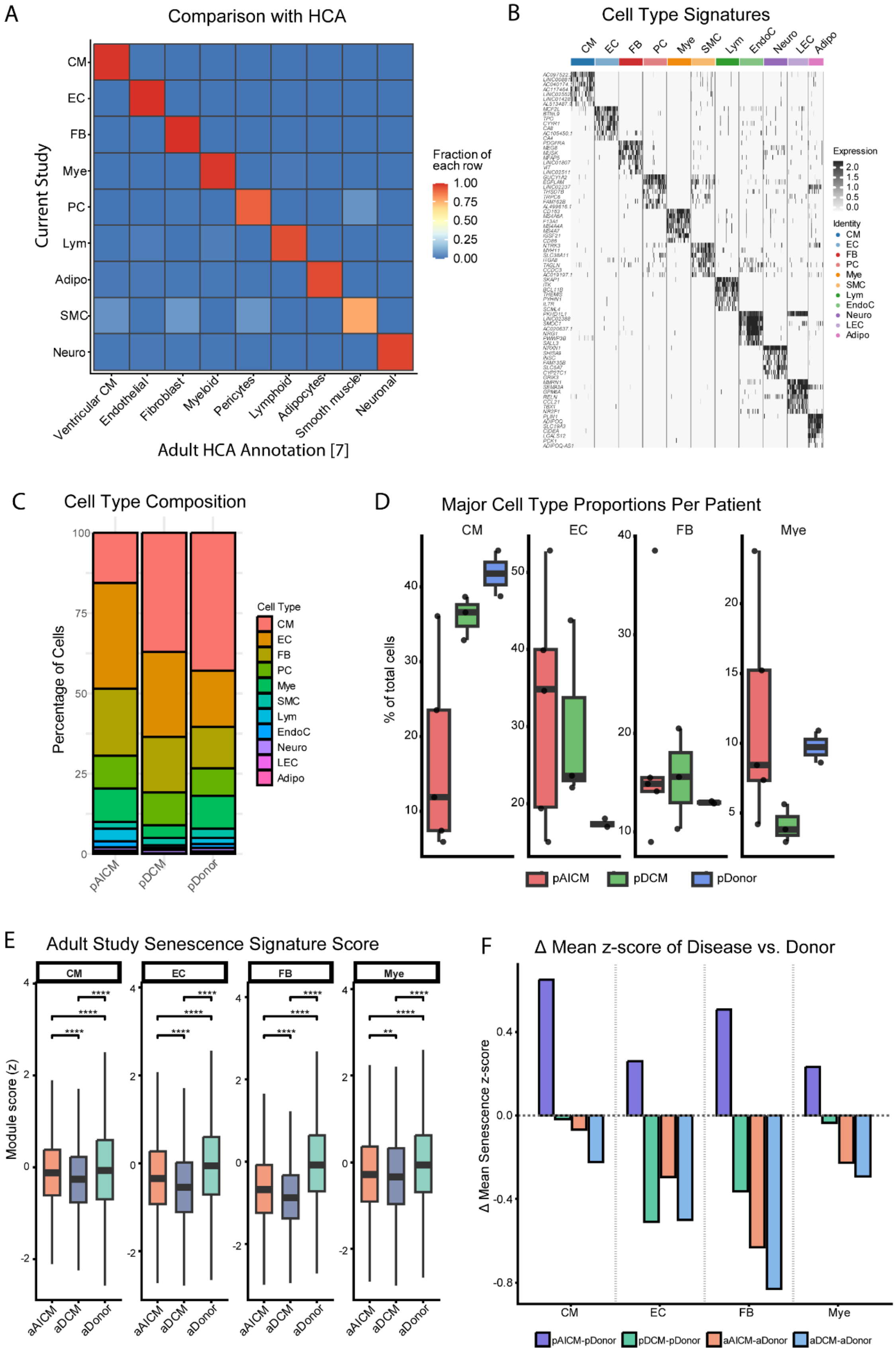

A
Comparison with HCA
Current Study
CM
EC
FB
Mye
PC
Lym
Adipo
SMC
Neuro
Ventricular CM
Endothelial
Fibroblast
Myeloid
Pericytes
Lymphoid
Adipocytes
Smooth muscle
Neuronal
Adult HCA Annotation [7]
Fraction of each row
1.00
0.75
0.50
0.25
0.00
B
Cell Type Signatures
CM
EC
FB
PC
Mye
SMC
Lym
EndoC
Neuro
LEC
Adipo
Expression
2.0
1.5
1.0
0.5
0.0
Identity
CM
EC
FB
PC
Mye
SMC
Lym
EndoC
Neuro
LEC
Adipo
C
Cell Type Composition
Percentage of Cells
100
75
50
25
0
pAICM
pDCM
pDonor
Cell Type
CM
EC
FB
PC
Mye
SMC
Lym
EndoC
Neuro
LEC
Adipo
D
Major Cell Type Proportions Per Patient
% of total cells
CM
EC
FB
Mye
pAICM
pDCM
pDonor
E
Adult Study Senescence Signature Score
Module score (z)
CM
EC
FB
Mye
****
**
aAICM
aDCM
aDonor
F
Δ Mean z-score of Disease vs. Donor
Δ Mean Senescence z-score
0.4
0.0
-0.4
-0.8
CM
EC
FB
Mye
pAICM-pDonor
pDCM-pDonor
aAICM-aDonor
aDCM-aDonor

Supplementary Figure 1: Validation of cell type annotation, and senescence scoring across pediatric and adult cardiomyopathy datasets. (A) Heatmap showing the fraction of cell clusters (rows) in the integrated snRNA-seq data predicted as specific cell types (columns) from a reference dataset. HCA: Human Cell Atlas (Litviňuková *et al.)*. **(B)** Heatmap of cell type signature gene expression (rows) in all cells, grouped by cell type (columns). Representative signature genes for each cell type are labeled on the left. Cardiomyocytes (CM), endothelial cells (EC), fibroblasts (FB), pericytes (PC), myeloid cells (Mye), smooth muscle cells (SMC), lymphoid cells (Lym), endocardial cells (EndoC), neuronal cells (Neuro), lymphatic endothelial cells (LEC) and adipocytes (Adipo). **(C)** Stacked bar plots showing the proportional composition of major cardiac cell types across pAICM, pDCM, and pDonor groups. **(D)** Box plots of per-patient cell type proportions for the four major cell compartments (CM, EC, FB, Mye) across pAICM, pDCM, and pDonor groups. Each point represents an individual patient sample. **(E)** Box plots of senescence module scores (z-scored) for CM, EC, FB, and Mye cell types in adult AICM (aAICM), adult DCM (aDCM), and adult donor (aDonor) samples, generated by applying the senescence gene signature to a published adult cardiomyopathy dataset (Guo et al.). (Wilcoxon rank-sum test with Benjamini-Hochberg FDR correction within each cell type; NS, not significant; *$P < 0.05$; **$P < 0.01$; ***$P < 0.001$; ****$P < 0.0001$). **(F)** Bar plot of the delta (Δ) mean senescence z-score for disease versus donor within each study, after normalization to the respective donor mean. Comparisons shown for pAICM vs. pDonor, pDCM vs. pDonor, aAICM vs. aDonor, and aDCM vs. aDonor across CM, EC, FB, and Mye compartments. Positive values indicate elevated senescence relative to donor controls.

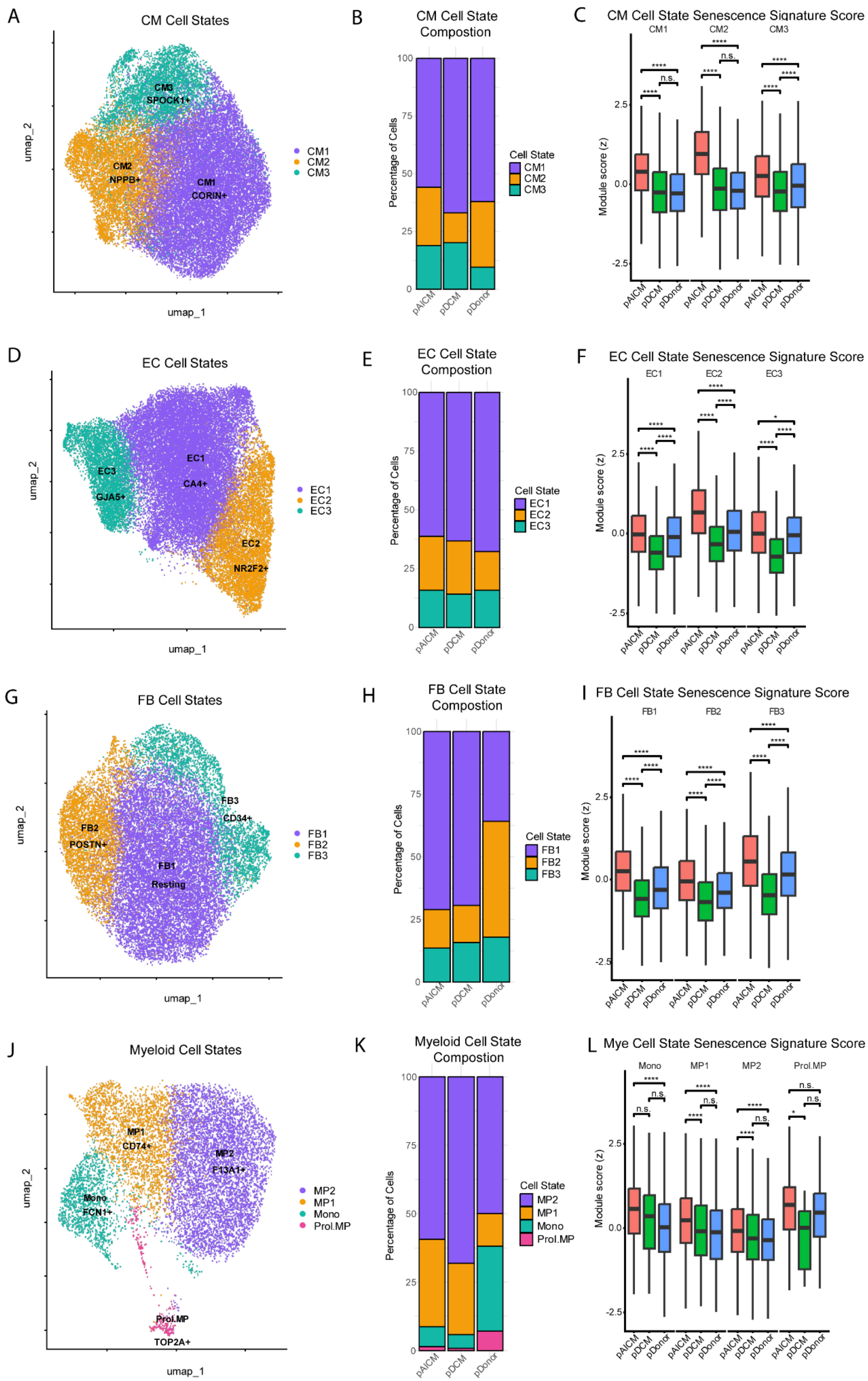

A
CM Cell States
CM3
SPOCK1+
CM2
NPPB+
CM1
CORIN+
umap_2
umap_1
CM1
CM2
CM3
B
CM Cell State Compostion
Percentage of Cells
100
75
50
25
0
pAICM
pDCM
pDonor
Cell State
CM1
CM2
CM3
C
CM Cell State Senescence Signature Score
CM1
CM2
CM3
Module score (z)
2.5
0.0
-2.5
****
n.s.
D
EC Cell States
EC3
GJA5+
EC1
CA4+
EC2
NR2F2+
EC1
EC2
EC3
E
EC Cell State Compostion
Cell State
F
EC Cell State Senescence Signature Score
*
G
FB Cell States
FB3
CD34+
FB2
POSTN+
FB1
Resting
FB1
FB2
FB3
H
FB Cell State Compostion
I
FB Cell State Senescence Signature Score
J
Myeloid Cell States
MP1
CD74+
MP2
F13A1+
Mono
FCN1+
Prol.MP
TOP2A+
MP2
MP1
Mono
Prol.MP
K
Myeloid Cell State Compostion
L
Mye Cell State Senescence Signature Score

Supplementary Figure 2. Cell state annotation and senescence scoring across cardiac cell type subclusters. (A) UMAP of cardiomyocyte (CM) subclusters identifying three cell states: CM1 (CORIN+, resting), CM2 (NPPB+), and CM3 (SPOCK1+). **(B)** Stacked bar plots showing the proportional composition of CM cell states (CM1, CM2, CM3) across pAICM, pDCM, and pDonor groups. **(C)** Box plots of senescence module scores (z-scored) for CM1, CM2, and CM3 cell states across pAICM, pDCM, and pDonor groups. **(D)** UMAP of endothelial cell (EC) subclusters identifying three cell states: EC1 (CA4+, capillary), EC2 (NR2F2+, venous), and EC3 (GJA5+, arterial). **(E)** Stacked bar plots showing the proportional composition of EC cell states across pAICM, pDCM, and pDonor groups. **(F)** Box plots of senescence module scores (z-scored) for EC1, EC2, and EC3 cell states across pAICM, pDCM, and pDonor groups. **(G)** UMAP of fibroblast (FB) subclusters identifying three cell states: FB1 (Resting), FB2 (POSTN+), and FB3 (CD34+). **(H)** Stacked bar plots showing the proportional composition of FB cell states across pAICM, pDCM, and pDonor groups. **(I)** Box plots of senescence module scores (z-scored) for FB1, FB2, and FB3 cell states across pAICM, pDCM, and pDonor groups. **(J)** UMAP of myeloid cell subclusters identifying four cell states: MP2 (F13A1+), MP1 (CD74+), Mono (FCN1+), and Prol.MP (TOP2A+). **(K)** Stacked bar plots showing the proportional composition of myeloid cell states across pAICM, pDCM, and pDonor groups. **(L)** Box plots of senescence module scores (z-scored) for MP2, MP1, Mono, and Prol.MP cell states across pAICM, pDCM, and pDonor groups. (For all box plots, statistical comparisons were performed using the Wilcoxon rank-sum test with Benjamini-Hochberg FDR correction; NS, not significant; *$P < 0.05$; **$P < 0.01$; ***$P < 0.001$; ****$P < 0.0001$.)

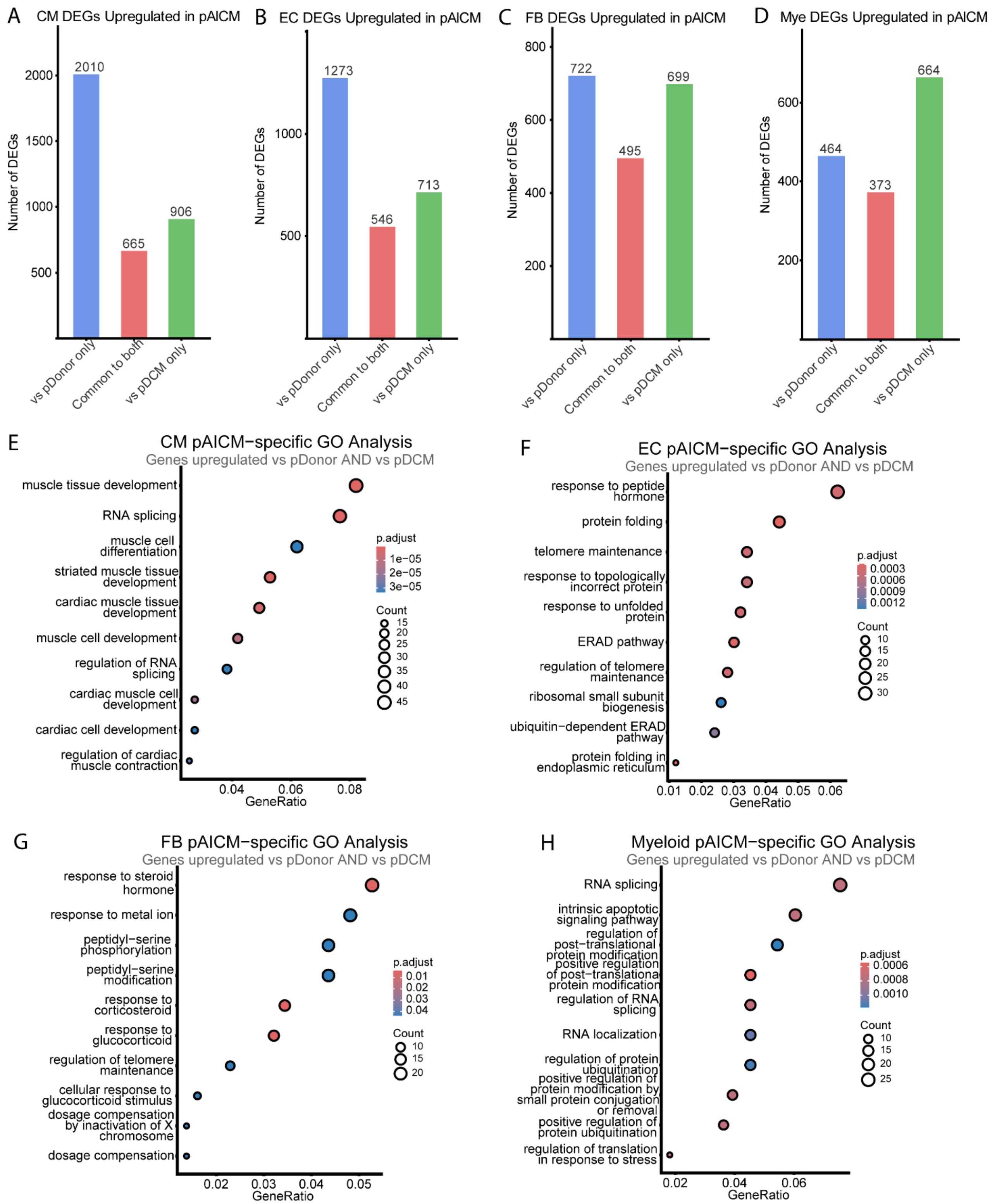
A
CM DEGs Upregulated in pAICM
B
EC DEGs Upregulated in pAICM
C
FB DEGs Upregulated in pAICM
D
Mye DEGs Upregulated in pAICM
Number of DEGs
vs pDonor only
Common to both
vs pDCM only
2010
665
906
1273
546
713
722
495
699
464
373
664
E
CM pAICM−specific GO Analysis
Genes upregulated vs pDonor AND vs pDCM
muscle tissue development
RNA splicing
muscle cell differentiation
striated muscle tissue development
cardiac muscle tissue development
muscle cell development
regulation of RNA splicing
cardiac muscle cell development
cardiac cell development
regulation of cardiac muscle contraction
p.adjust
Count
GeneRatio
F
EC pAICM−specific GO Analysis
Genes upregulated vs pDonor AND vs pDCM
response to peptide hormone
protein folding
telomere maintenance
response to topologically incorrect protein
response to unfolded protein
ERAD pathway
regulation of telomere maintenance
ribosomal small subunit biogenesis
ubiquitin−dependent ERAD pathway
protein folding in endoplasmic reticulum
G
FB pAICM−specific GO Analysis
Genes upregulated vs pDonor AND vs pDCM
response to steroid hormone
response to metal ion
peptidyl−serine phosphorylation
peptidyl−serine modification
response to corticosteroid
response to glucocorticoid
regulation of telomere maintenance
cellular response to glucocorticoid stimulus
dosage compensation by inactivation of X chromosome
dosage compensation
H
Myeloid pAICM−specific GO Analysis
Genes upregulated vs pDonor AND vs pDCM
RNA splicing
intrinsic apoptotic signaling pathway
regulation of post−translational protein modification
positive regulation of post−translational protein modification
regulation of RNA splicing
RNA localization
regulation of protein ubiquitination
positive regulation of protein modification by small protein conjugation or removal
positive regulation of protein ubiquitination
regulation of translation in response to stress

Supplementary Figure 3. Cell type-specific differentially expressed genes (DEGs) upregulated in pediatric anthracycline-induced cardiomyopathy (pAICM) and pathway enrichment analysis. (A, B, C, D) Bar plots showing the number of DEGs significantly upregulated in pAICM (adjusted $P < 0.05$, $log2FC > 0.25$) for cardiomyocytes (CM), endothelial cells (EC), fibroblasts (FB), and myeloid cells (Mye), respectively. Genes are stratified as: unique to pAICM vs. pDonor (blue), common to both comparisons (red), and unique to pAICM vs. pDCM (green). Common genes represent pAICM-specific upregulation shared across both disease comparisons. **(E, F, G, H)** Gene Ontology (GO) biological process enrichment analysis of pAICM-specific upregulated genes (common to both comparisons) for CM, EC, FB, and Mye, respectively. Dot size represents the number of genes contributing to each term (Count); dot color reflects the adjusted p-value (BH correction). Top 10 enriched terms are shown ranked by GeneRatio.

Supplementary References

1. Belger C, Abrahams C, Imamdin A, et al. Doxorubicin-induced cardiotoxicity and risk factors. *Int J Cardiol Heart Vasc.* 2024;50:101332.
2. Puri K, Denfield SW, Adachi I, et al. Ventricular assist device support for children with chemotherapy-induced cardiomyopathy and advanced heart failure: Perspectives gained from a single-center experience. *Pediatr Transplant.* 2022;26(5):e14286.
3. Koenig AL, Shchukina I, Amrute J, et al. Single-cell transcriptomics reveals cell-type-specific diversification in human heart failure. *Nat Cardiovasc Res.* 2022;1(3):263-80.
4. Hill MC, Kadow ZA, Long H, et al. Integrated multi-omic characterization of congenital heart disease. *Nature.* 2022;608(7921):181-91.
5. Li X, Turaga D, Li RG, et al. The Macrophage Landscape Across the Lifespan of a Human Cardiac Allograft. *Circulation.* 2024;149(21):1650-66.
6. Wolock SL, Lopez R, and Klein AM. Scrublet: Computational Identification of Cell Doublets in Single-Cell Transcriptomic Data. *Cell Syst.* 2019;8(4):281-91 e9.
7. Litvinukova M, Talavera-Lopez C, Maatz H, et al. Cells of the adult human heart. *Nature.* 2020;588(7838):466-72.
8. Tirosh I, Izar B, Prakadan SM, et al. Dissecting the multicellular ecosystem of metastatic melanoma by single-cell RNA-seq. *Science.* 2016;352(6282):189-96.
9. Saul D, Kosinsky RL, Atkinson EJ, et al. A new gene set identifies senescent cells and predicts senescence-associated pathways across tissues. *Nat Commun.* 2022;13(1):4827.
